# Metabolomic Profiles in Childhood and Adolescence Are Associated with Fetal Overnutrition

**DOI:** 10.3390/metabo12030265

**Published:** 2022-03-19

**Authors:** Ellen C. Francis, Katerina Kechris, Catherine C. Cohen, Gregory Michelotti, Dana Dabelea, Wei Perng

**Affiliations:** 1 Lifecourse Epidemiology of Adiposity and Diabetes (LEAD) Center, University of Colorado Denver Anschutz Medical Campus, Aurora, CO 80045, USA; catherine.cioffi@cuanschutz.edu (C.C.C.); dana.dabelea@cuanschutz.edu (D.D.); wei.perng@cuanschutz.edu (W.P.); 2 Department of Biostatistics and Informatics, Colorado School of Public Health, University of Colorado Anschutz Medical Campus, Aurora, CO 80045, USA; Katerina.Kechris@cuanschutz.edu; 3 Department of Pediatrics, School of Medicine, University of Colorado Anschutz Medical Campus, Aurora, CO 80045, USA; 4 Metabolon, Inc., Morrisville, NC 27560, USA; GMichelotti@metabolon.com; 5 Department of Epidemiology, Colorado School of Public Health, University of Colorado Denver Anschutz Medical Campus, Aurora, CO 80045, USA

**Keywords:** pregnancy, obesity, gestational diabetes mellitus, metabolomics, childhood

## Abstract

Fetal overnutrition predisposes offspring to increased metabolic risk. The current study used metabolomics to assess sustained differences in serum metabolites across childhood and adolescence among youth exposed to three typologies of fetal overnutrition: maternal obesity only, gestational diabetes mellitus (GDM) only, and obesity + GDM. We included youth exposed in utero to obesity only (BMI ≥ 30; *n* = 66), GDM only (*n* = 56), obesity + GDM (*n* = 25), or unexposed (*n* = 297), with untargeted metabolomics measured at ages 10 and 16 years. We used linear mixed models to identify metabolites across both time-points associated with exposure to any overnutrition, using a false-discovery-rate correction (FDR) <0.20. These metabolites were included in a principal component analysis (PCA) to generate profiles and assess metabolite profile differences with respect to overnutrition typology (adjusted for prenatal smoking, offspring age, sex, and race/ethnicity). Fetal overnutrition was associated with 52 metabolites. PCA yielded four factors accounting for 17–27% of the variance, depending on age of measurement. We observed differences in three factor patterns with respect to overnutrition typology: sphingomyelin-mannose (8–13% variance), skeletal muscle metabolism (6–10% variance), and 3-carboxy-4-methyl-5-propyl-2-furanpropanoic acid (CMPF; 3–4% variance). The sphingomyelin-mannose factor score was higher among offspring exposed to obesity vs. GDM. Exposure to obesity + GDM (vs. GDM or obesity only) was associated with higher skeletal muscle metabolism and CMPF scores. Fetal overnutrition is associated with metabolic changes in the offspring, but differences between typologies of overnutrition account for a small amount of variation in the metabolome, suggesting there is likely greater pathophysiological overlap than difference.

## 1. Introduction

Maternal obesity and Gestational Diabetes Mellitus (GDM) are interrelated metabolic conditions leading to fetal overnutrition, a term that refers to fetal exposure to excess maternal fuels including but not limited to glucose, amino acids, and lipids [1]. Although these conditions are interrelated, GDM with or without maternal obesity may represent different metabolic entities [2], and thus may have implications for in utero programming. For instance, large epidemiological studies have demonstrated independent and exposure-specific effects of GDM or obesity in relation to offspring outcomes (e.g., obesity vs. fat mass % vs. cardiovascular disease vs. diabetes) [1,3,4,5,6]. In 597 adult offspring of Danish women with GDM, there was an eight-fold increased risk of pre-diabetes or diabetes [3]. Among over 4500 mother–offspring pairs from an international multicenter cohort, GDM has been associated with a 5% risk difference in offspring obesity during adolescence, even after accounting for maternal body mass index (BMI) [4]. In a Swedish population-based cohort, age-adjusted cardiovascular disease rates in offspring increased linearly with maternal BMI status in pregnancy [5].

Recent data indicate that there are overlapping as well as distinct changes in the metabolome of offspring [7,8,9], depending on exposure to these fetal overnutrition typologies (i.e., obesity or GDM). These studies have used metabolomics in offspring to capture the consequences of exposure to maternal obesity or GDM. For instance, maternal overweight/obesity, but not glucose tolerance status, has been associated with increased branched-chain amino acids (BCAAs) in cord blood [7,10,11]. Maternal BMI and glucose levels have been correlated with an offspring metabolite profile characterized by phospho- and sphingo-lipids in childhood (5–7 years of age) [8]. Similarly, we have found that exposure to GDM was associated with an offspring metabolite profile characterized by higher phospholipids during childhood and adolescences [9]. However, this association was attenuated after accounting for maternal pre-pregnancy BMI, which may represent an overlapping biological pathway [9]. This biological overlap is likely related to the effects of excess adiposity and insulin resistance, leading to increased glucose levels during pregnancy [12]. Nevertheless, there may also be distinct biochemical/metabolic pathways affected by exposure to maternal obesity and/or GDM. A more nuanced characterization of such differences with respect to type of fetal overnutrition—i.e., exposure to maternal obesity only, GDM only, or both—may elucidate differences in metabolic processes linking these conditions to long-term offspring health.

The objective of this hypothesis-generating analysis was to extend current knowledge on the relationship between fetal overnutrition and offspring metabolic health by leveraging repeated untargeted metabolomics data in offspring across 6 years of follow-up. We aimed to: (1) identify a set of metabolites associated with any type of fetal overnutrition; (2) identify differences in offspring metabolite profiles with respect to maternal obesity only, GDM only, or both, thereby shedding light on distinct and shared pathways, and (3) assess the correlation of offspring metabolite profiles with conventional indicators of metabolic health to aid in interpretation of the metabolite profiles identified.

## 2. Results

### 2.1. Characteristics

The mean (±SD) age of participants at the childhood visit was 10.4 ± 1.5 years (range, 6.0–13.9 years), and at the adolescent visit, it was 16.7 ± 1.2 years (range, 12.6–19.6 years). Approximately half the participants were female, and 33% were exposed to any fetal overnutrition (obesity or GDM). Maternal and child characteristics by fetal overnutrition typology are presented in Table 1. The frequency of women who smoked during pregnancy differed across typology: of the women with obesity and GDM, 20.0% reported smoking, whereas the percentage of smokers among women with GDM only, obesity only, or neither was 17.9%, 6.1%, and 7.2%, respectively. There was a higher percentage of Hispanic offspring among women with obesity (57.6%), and a higher percentage of non-Hispanic White offspring among women with GDM only or obesity and GDM (71.4% and 52.0%). As expected, offspring of women with obesity and GDM had a higher birthweight-for-gestational-age z-score. At each research visit, offspring of women with either obesity or GDM tended to be younger. Offspring of women with obesity only had the highest mean BMI.

### 2.2. Identification of Metabolites in Offspring Associated with Any Fetal Overnutrition

The metabolome-wide association study (MWAS) yielded 52 metabolites across childhood and adolescence that were significantly associated with exposure to any fetal overnutrition (obesity or GDM) versus no exposure to overnutrition (false-discovery-rate [FDR] *p*-value < 0.20) (Table 2). These metabolites were predominately from peptide, amino acid, and lipid super classes.

### 2.3. Associations between Fetal Overnutrition Typologies and Offspring Metabolite Profiles

We implemented the PCA on the 52 metabolites identified from the MWAS and selected six factors to retain at each visit based on the break in Scree plot and shown in Table 3.

Following qualitative assessment of the top loading metabolites, we noted consistency in factor composition for four factors across the two visits: γ-glutamyl-peptide factor, sphingomyelin-mannose factor, skeletal muscle metabolism factor, and the 3-carboxy-4-methyl-5-propyl-2-furanpropanoic acid (CMPF) factor. The first factor, which accounts for the most variation in metabolites associated with fetal overnutrition (i.e., 44% variance at the childhood visit, 20% at the adolescent visit), was driven by compounds in the γ-glutamyl-peptide amino acid subclass (top three metabolites were: γ-glutamylglutamate γ-glutamyl-α-lysine, γ-glutamylglycine) was well as some phospholipids and fatty acids.

**Figure 1:**
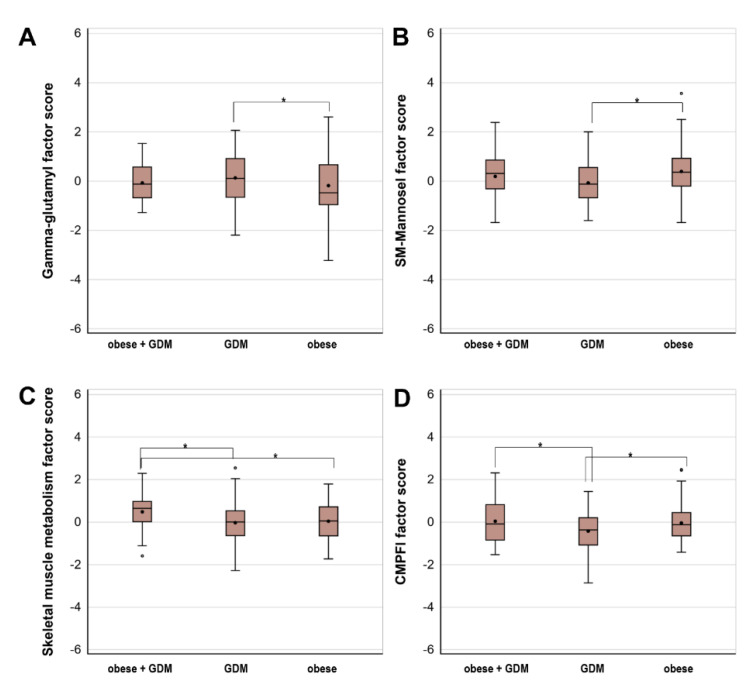
Unadjusted associations (β [95% CI]) of fetal overnutrition (obesity and GDM, obesity only, and GDM only) with metabolite factor scores across 6 years of follow-up among 444 youth in the EPOCH cohort. (**A**) Longitudinal association fetal overnutrition typology and γ-glutamyl factor scores: offspring of women with obesity only had significantly lower factor scores compared to offspring of women with GDM only. (**B**) Longitudinal association fetal overnutrition typology and sphingomyelin-mannose factor scores: offspring of women with obesity only had significantly higher factor scores compared to offspring of women with GDM only. (**C**) Longitudinal association fetal overnutrition typology and skeletal muscle metabolism factor scores: offspring of women with obesity and GDM had significantly higher factor scores compared to offspring of women with GDM only, and offspring of women with obesity only. (**D**) Longitudinal association fetal overnutrition typology and CMPF factor scores: offspring of women with obesity and GDM had significantly higher factor scores compared to offspring of women with GDM only. Offspring of women with obesity only had significantly higher factor scores compared to women with GDM only. Abbreviations: CMPF, 3-carboxy-4-methyl-5-propyl-2-furanpropanoic acid; GDM, Gestational Diabetes Mellitus; Sphingomyelin, SM; OB, pre-pregnancy obesity. * *p* < 0.05.

Although the γ-glutamyl-peptide factor score was lower among offspring of women with obesity only vs. GDM only (Figure 1), covariate adjustment attenuated the difference, and the confidence intervals included the null (Table 4).

We found that the other three factors did differ by type of fetal overnutrition in both unadjusted (Figure 1) and adjusted models (Table 4). The sphingomyelin-mannose factor score, which had a similar top loading metabolite composition at each visit despite a difference in factor order, was higher among offspring of women with obesity only vs. GDM only. The skeletal muscle metabolism factor score was higher among offspring of women with obesity and GDM vs. GDM only or obesity only. The CMPF factor score was higher among offspring of women with obesity and GDM or obesity only vs. GDM only. The associations of fetal overnutrition typology with key metabolites (i.e., factor loading > 0.40) within each of these factors are shown in Supplementary Table S1. In general, the direction and significance of associations with key metabolites are similar but smaller in magnitude to those of the overall metabolite factors.

#### Sensitivity Analyses

After excluding women with type-one diabetes, the estimates with GDM only as the reference were attenuated, but with no impact on statistical significance. After adjustment for offspring BMI (a potential mediator to the relationship between fetal overnutrition and later-life metabolite profiles), the difference in the sphingomyelin-mannose factor scores between offspring of women with obesity only vs. GDM only was attenuated to null (Supplementary Table S2). No other associations were impacted by adjustment for offspring BMI. There were minimal changes in the significance of findings after adjusting for offspring Tanner stage, kilocalories, physical activity, or birthweight-for-gestational-age z-score (Supplementary Table S2). Adjusting for GDM treatment modality had minimal impact on the magnitude of association and significance, with the greatest impact (6% reduction in beta) observed for differences in the skeletal muscle metabolism factor between obesity and GDM vs. obesity only. There was no statistical evidence that the association between fetal overnutrition and metabolite profiles/factors scores were different in males versus females (all *p*-values for interaction terms >0.1)

### 2.4. Correlation of Offspring Metabolite Profiles and Indicators of Metabolic Health

**Figure 2:**
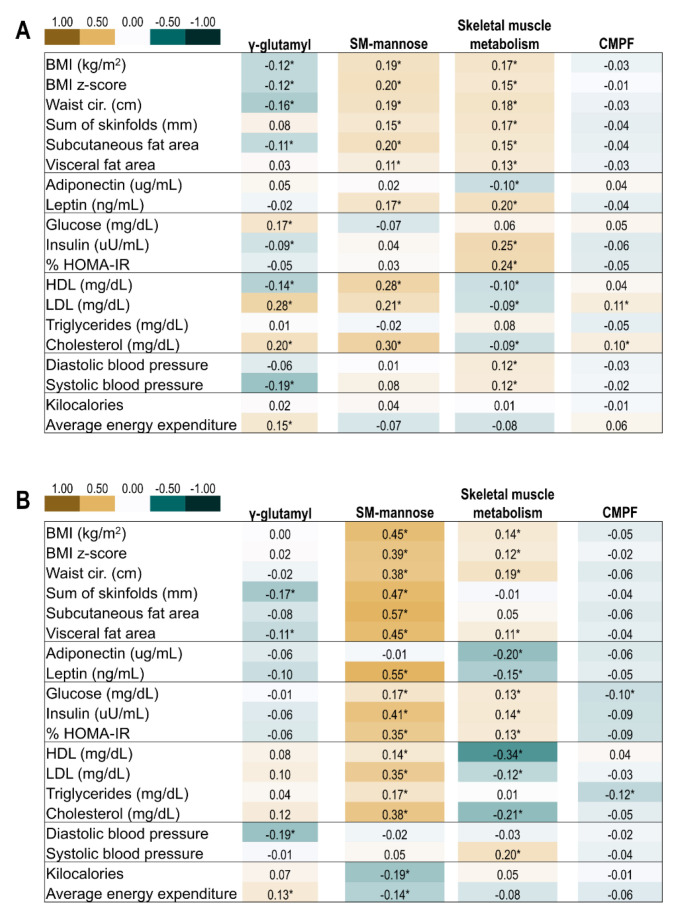
Pearson correlation of offspring clinical metabolic and body composition markers and metabolomic factors at the childhood and adolescent visits. * *p* < 0.05. (**A**) Correlations at ~10 years of age. (**B**) Correlations at ~16 years of age. Abbreviations: CMPF, 3-carboxy-4-methyl-5-propyl-2-furanpropanoic acid; SM, sphingomyelins.

Pearson correlations of the four metabolite factors of interest with conventional metabolic biomarkers and body composition are shown in Figure 2. In general, a higher score for the γ-glutamyl-peptide factor correlated with lower measures of adiposity, higher total cholesterol, and greater physical activity. The sphingomyelin-mannose factor was strongly positively correlated with adiposity and lipids (high-density lipoprotein, low-density lipo-protein, cholesterol). The skeletal muscle metabolism factor was positively correlated with adiposity and insulin resistance, and inversely correlated with lipids. The CMPF factor showed weak to no correlations with offspring indicators of metabolic health.

## 3. Discussion

### 3.1. Summary of Overall Findings

In this longitudinal study of 440 mother–offspring pairs, we sought to identify sustained differences in serum metabolites across childhood and adolescence among youth exposed to fetal overnutrition, and explored differences in metabolite profiles with respect to typology of fetal overnutrition (obesity only, GDM only, obesity and GDM). Exposure to any fetal overnutrition was associated with persistent metabolic changes in the offspring, but further differences between typologies of overnutrition account for a relatively small amount of variation in the metabolome, suggesting that there is likely a greater degree of pathophysiological overlap than distinct differences.

### 3.2. γ-Glutamyl Peptides Factor

The greatest amount of variation in metabolites associated with developmental overnutrition was accounted for by the γ-glutamyl-peptide factor, although this factor did not differ by overnutrition typology after adjustment for confounders. Key metabolites in this factor were derivates of glutamate (non-essential amino acid), lysine (essential amino acid), and glycine (gluconeogenic amino acid). GDM has been associated with a higher glutamine/glutamate ratio in cord blood [10] and glutamine has been associated with amino acid-mediated insulin secretion, and is sensitive to dietary changes [15,16,17]. Thus, this latter finding in cord blood may reflect both offspring’s exposure to hyperglycemia and the dietary and lifestyle changes implemented by women with GDM, which are also likely adopted by their children.

Methionine sulfoxide was also a top loading metabolite in the γ-glutamyl-peptide factor. This amino acid metabolite is a resulting compound of oxidation by reactive oxygen species that influences redox homeostasis and regulates many metabolic pathways including protein synthesis [18]. In mice with diet-induced obesity, deletion of enzymes required for reduction of methionine sulfoxide resulted in diminished insulin receptor function [19], highlighting a potential role of protein oxidation in insulin signaling. Thus, this component of the γ-glutamyl-peptide factor may represent differences in oxidative stress associated with fetal overnutrition.

Interestingly, we found that the correlation of the γ-glutamyl-peptide factor with indicators of metabolic health in offspring was in a direction indicative of a more favorable metabolic profile—lower adiposity, increased insulin sensitivity, and greater physical activity. In children, plasma levels of glutamine, a precursor to glutamate, were higher following a reduction in BMI [15]. Indeed, our finding that γ-glutamyl-peptide factor metabolites were associated with both exposure to overnutrition and a more favorable metabolic profile in offspring may simply reflect the precursor–product relationship of glutamine and glutamate, and that some of these youth adopted healthier behaviors.

### 3.3. Sphingomyelin-Mannose Factor

Although there was no unifying theme among the metabolites in this factor, sub-classes of metabolites in this pattern have been linked to obesity and metabolic risk [20,21,22]. Sphingomyelins and dihydrosphongomyelin are important constituents of plasma membranes that interact closely with cholesterol and directly impact cholesterol homeostasis [23]. Indeed, we found that the sphingomyelin-mannose factor was more strongly correlated with cholesterol in childhood and adolescence compared to any other factor. We found a higher sphingomyelin-mannose factor score among offspring of women with obesity only vs. GDM only, which was likely driven by offspring adiposity, as the estimate was attenuated after adjusting for offspring BMI and this factor was strongly correlated with concurrent subcutaneous and visceral fat.

Interpretation of our findings with this factor in the context of previous studies is somewhat difficult given that prior studies have used different indicators of fetal overnutrition (maternal weight gain, BMI, glucose levels, GDM), and metabolite profile composition and choice of targeted assays has been study-specific. In a pre-birth cohort of 330 mother–offspring pairs with metabolomics measured in childhood (5–7 years of age), a metabolite profile characterized by phosphatidylcholines, plasmalogens, sphingomyelins and some ceramides was positively correlated with maternal pregnancy weight gain, but negatively correlated with fasting glucose [8]. These findings remained significant following adjustment for maternal waist circumference—a marker of central adiposity. However, among 412 mother–offspring pairs, GDM was positively associated with cord blood metabolites from sub-classes of phosphatidylcholines and sphingomyelins, but these differences were no longer significant after adjusting for maternal BMI [24]. Taken together, the associations of fetal overnutrition with phospholipids and sphingomyelins in offspring may represent pathways of adiposity and glycemia that are distinct and specific to the lipid class, as well as overlapping, and which operate through shared maternal–offspring risk of obesity.

### 3.4. Skeletal Muscle Metabolism Factor

Key compounds in this factor included α-hydroxyisocaproate and 2-hydroxy-3-methylvalerate, ketoacid metabolites of the branched-chain amino acids (BCAA) leucine and isoleucine; malate and citrate, TCA cycle intermediaries [25]; and urate, a catabolite of purine metabolism [26]. Skeletal muscle metabolism of BCAAs is critical for maintaining energy homeostasis, as well as anaplerotic supply to the TCA cycle. In the current study, we found that exposure to any fetal overnutrition was associated with a higher score for this factor, especially among those exposed to obesity and GDM compared to either typology alone. Further, in both childhood and adolescence, the skeletal muscle metabolism factor was correlated with greater adiposity and HOMA-IR. In general, maternal BMI/obesity has been positively associated with cord blood BCAAs and their metabolites [7,10,11], which in turn, have been positively related to birthweight [7,11]. These data point toward the early origins of the relationship between obesity, BCAA catabolism, and insulin resistance, which has been repeatedly found in animal and adult populations [27,28,29,30].

### 3.5. CMPF Factor

CMPF and its hydroxylated metabolite hydroxy-CMPF are metabolites of furan and long-chain omega-3 fatty acids [31,32]. Although data have linked CMPF to diabetes and β-cell dysfunction, this finding was attributed to differences in dietary intake and not directly implicated in glucose metabolism [33,34]. We found that offspring of women with obesity and GDM or obesity only had higher CMPF factor scores compared to offspring exposed to GDM only, suggesting an association specific to maternal obesity. However, the CMPF factor accounted for only 3–4% of the variation in the metabolites associated with any developmental overnutrition and was not strongly correlated with conventional indicators of metabolic health in offspring. To our knowledge, prior studies in youth have not identified associations of maternal glucose or adiposity with CMPF. Thus, in the context of fetal overnutrition, the relevance of the differences in the CMPF factor requires further investigation.

### 3.6. Strengths and Limitations

Our study has several strengths. First, the repeated metabolomics data are a unique asset of the EPOCH cohort that allowed us to examine the association of fetal overnutrition across two sensitive life stages for development of metabolic disease risk: childhood and adolescence [6]. Assessment of sustained differences in metabolite profiles across these life stages has important implications for chronic disease etiology, which typically take root during the first decade of life [35,36], can be tracked across development [37], and eventually manifest as overt chronic disease in adulthood [38]. Second, we used a multi-step analytical approach that identified individual metabolites, as well as correlated metabolites depicted by a data-driven latent construct, enabling us to capture the biochemical interactions among compounds on the same and/or related metabolic pathways.

Limitations include only having data on GDM diagnosis (yes/no) and pre-pregnancy BMI, without further detail on specifics of maternal glycemic physiology (e.g., insulin secretion, insulin resistance). This may have contributed to the minimal differences in fetal overnutrition typology detected, as these features have been related to specific differences in neonatal outcomes [39]. Although we had a relatively large sample size of offspring (*n* = 440), especially in comparison to other metabolomics analyses of intrauterine exposures and outcomes in youth (*n* for most <350) [40,41], some fetal overnutrition-exposure groups were relatively small (e.g., obesity and GDM), which may have impacted our power to detect specific contrasts with this group. It is also worth noting that the PCA was implemented separately for the childhood and adolescence metabolite data. Thus, while the composition of top-loading metabolites for the factors of interest were similar, the weighting scheme for the factor loadings differed slightly between time points, potentially hindering direct comparability of factors across the follow-up. However, this approach is widely used in nutritional epidemiology to identify distinct dietary patterns both longitudinally within the same population, as well as for comparability purposes across populations [42,43,44]. Future longitudinal studies with complex matrix-variate data, similar to repeated measures of high-dimensional data, might consider the use of novel two-way principal component methods [45]. Although the current study was hypothesis-generating, it is worthwhile to note that the concurrent measurement of conventional metabolic biomarkers and metabolomics hinders directionality and makes any inference on causal mechanisms challenging. Finally, given the large number of metabolites used in the MWAS approach, we cannot discount the potential for false-positive findings, though we applied an (FDR) correction and implemented dimension reduction to reduce this possibility.

### 3.7. Conclusion and Future Direction

In this study of 444 mother–child pairs, fetal overnutrition, defined as in utero exposure to maternal obesity, GDM or both, was associated with differences in fasting serum concentrations of 52 metabolites in offspring across childhood and adolescence after FDR correction. Further assessment of differences in metabolite profiles within typology of fetal overnutrition revealed differences in sphingomyelin-mannose, skeletal muscle metabolism, and CMPF metabolite profiles. However, these differences accounted for a relatively small percent of variation in the metabolomics dataset, suggesting that although maternal obesity and GDM are often regarded as distinct conditions, their impact on the offspring metabolome does not differ greatly. Thus, studies investigating the impacts of obesity and GDM on offspring metabolite profiles may consider maternal obesity and GDM not as separate entities, but rather as degrees of severity within a metabolic spectrum. The metabolite factors in this study were correlated with established indicators of adiposity and metabolic risk in offspring, and thus, may capture some of the underlying metabolic dysregulation and chronic disease risk associated with fetal overnutrition [3,4,5]. Given the growing evidence that fetal overnutrition has impacts on offspring health across the life span, future research is warranted to identify whether etiologic pathways, mechanisms, and mediators linking fetal overnutrition to the metabolic differences found herein are relevant to overt disease states.

## 4. Materials and Methods

### 4.1. Study Population

This hypothesis-generating analysis included mother–offspring pairs from the Exploring Perinatal Outcomes among Children (EPOCH) cohort. Eligible participants were children exposed to maternal GDM and a random sample of children not exposed and without intrauterine growth restriction (defined as birthweight-for-gestational-age score <the 10th percentile) (*n* = 604). Eligibility criteria for EPOCH were offspring of singleton pregnancies delivered between 1992 and 2002 whose biological mothers were members of the Kaiser Permanente of Colorado Health Plan. Details on recruitment and study population have been previously published [46]. In 2006–2009 and 2012–2015, offspring were invited to complete two research visits: first in childhood between 6 and 14 years old (mean age, 10.4 ± 1.5 years) and second in adolescence between 12 and 19 years old (mean age, 16.7 ± 1.2 years). From here forward, these visits are referred to as the childhood and adolescent visits, respectively. At both research visits, fasting blood was collected, refrigerated immediately, processed within 24 h, and stored at −80 °C until the time of analysis. These samples were used for untargeted metabolomics profiling and conventional biomarker assays.

For the current analysis, we excluded women missing data on pre-pregnancy BMI (*n* = 161), followed by offspring without sufficient blood volume for untargeted metabolomics profiling (*n* = 3). The analytic sample for this study included 440 mother–offspring pairs. In comparison to the 160 offspring who were not included in this analysis, the present sample was slightly younger and had a lower proportion of females, lower proportion of non-Hispanic white, and higher proportion of Hispanic youth.

### 4.2. Assessment of Exposure to Fetal Overnutrition

#### 4.2.1. Gestational Diabetes Mellitus

Women were screened for GDM based on the National Diabetes Data Group, which follows a two-step approach [47]. Presence of a GDM diagnosis was abstracted from medical records.

#### 4.2.2. Obesity

Maternal pre-pregnancy BMI (kg/m^2^) was calculated from pre-pregnancy weight abstracted from the medical records and height measured at the childhood visit. Obesity was defined as a pre-pregnancy BMI ≥ 30.0 kg/m^2^.

#### 4.2.3. Typology of Fetal Overnutrition

We assessed three typologies of fetal overnutrition: resulting from both maternal obesity and GDM (*n* = 25), maternal obesity only (*n* = 66), and GDM only (*n* = 56). Offspring of women without obesity or GDM were the reference group (*n* = 297).

### 4.3. Assessment of Metabolite Profiles in Offspring

Details on the untargeted metabolomic profiling in the EPOCH cohort have been published [9,48]. Briefly, Metabolon © (Morrisville, NC, USA) carried out untargeted metabolomics from the fasting serum collected at the childhood and adolescent visits using a multi-platform mass spectroscopy (MS)-based technique. Serum samples from both visits were analyzed at the same time, resulting in balanced batches and increased comparability of relative metabolite concentrations across both time points. Prior to formal statistical analysis, we removed metabolites with ≥20% missing values and imputed metabolites with <20% missing using the *k*-nearest neighbor technique (*k* = 10). There were 766 metabolites identified in both batches from the childhood and adolescent visit. Metabolite levels were log10-transformated, normalized, and corrected for batch effects (as well as other biological and technical variability) using the remove-unwanted-variation method (the number of factors of unwanted variation estimated from the data [*k*] = 2). In this analysis, we retained 637 metabolites at both the childhood and adolescent visit that were annotated. All metabolite processing was performed using R (Version 3.5.3; Vienna, Austria).

### 4.4. Assessment of Conventional Biomarkers of Metabolic Risk in Offspring

#### 4.4.1. Biomarkers

Fasting triglycerides (TGs), total cholesterol, high-density lipoprotein (HDL), low-density lipoprotein (LDL), and glucose were measured using enzymatic kits. Insulin was measured using a radioimmune assay, and leptin and adiponectin were measured using a Multiplex assay kit, all by Millipore Corporation (Darmstadt, Germany). We calculated the Homeostatic Model Assessment of Insulin Resistance (HOMA-IR). At both research visits, research assistants measured offspring’ blood pressure twice in the sitting position using an oscillometric monitor (Dinamap ProCare V100).

#### 4.4.2. Anthropometric and Body Composition

At each research visit, offspring waist circumference, triceps and subscapular skinfold thickness, height, and weight were measured. BMI was calculated as kg/m^2^, age- and sex-specific BMI z-scores were derived using the World Health Organization (WHO) growth reference for children aged 5–19 years [49], and the mean of triceps and subscapular skinfolds were summed. MRI of the abdominal region was used to quantify visceral adipose tissue (VAT) and subcutaneous adipose tissue (SAT) depots with a 3 T HDx Imager (General Electric, Waukashau, WI, USA) by a trained technician. One axial, 10 mm, T1-weighted image, at the umbilicus or L4/L5 vertebrae, was analyzed to determine SAT and VAT content by a single reader, blinded to exposure status.

#### 4.4.3. Lifestyle Behaviors

At each research visit, total energy intake (calories/day) was assessed using the Block Kid’s Food Questionnaire [50]. Average energy expenditure was calculated based on number of minutes of moderate-to-vigorous activity per day based on a 3-day self-report physical activity questionnaire [51].

### 4.5. Covariates

Maternal level of education, total household income, and smoking at any time during pregnancy were self-reported during the childhood visit. Offspring race and ethnicity, sex, and date of birth were self-reported at the first research visit.

### 4.6. Statistical Analysis

We assessed bivariate associations of maternal and offspring characteristics with typology of fetal overnutrition and tested for statistical differences using an ANOVA for continuous variables and Pearson chi-squared tests for categorical variables. This step, in conjunction with prior knowledge of determinants of metabolic health in youth, informed covariate selection for multivariable analysis. We then conducted the analysis in three sequential steps outlined below.

#### 4.6.1. Identification of Offspring Metabolites Associated with Any Fetal Overnutrition

We implemented a metabolome-wide association study (MWAS) using linear mixed models to identify offspring metabolites across the childhood and adolescent visits that were persistently associated with exposure to any type of fetal overnutrition.

Y_ij_ = β_1_x_1i_ + β_2_x_2i_ + X_3i_β_3_ + x_4ij_β_4_ + ε_ij_ (1)

where Y is the metabolite for individual i at time j. x_1_ = intercept; x_2_ = fetal overnutrition category (maternal obesity or GDM vs. neither); X_3_ = a vector of time-invariant factors (maternal smoking, offspring sex, ethnicity and race); x_4_ = offspring age for individual i at time j; ε = error term i at time j.

For these models, the outcome (Y) is repeated assessments of the metabolites for individual i at time j (childhood and adolescent visits), βs are the main independent variable/exposure of interest (fetal overnutrition: maternal obesity or GDM vs. neither), and covariates (maternal smoking during pregnancy, offspring sex, and ethnicity and race, and offspring age for individual i at time j). We included offspring ID as a repeated subject statement to account for correlation between metabolites from the same individual with an unstructured correlation matrix. Considering the number of tests and high degree of correlation among metabolites, we employed a FDR described by Benjamini and Hochberg (1995) at level α = 0.20 [52].

#### 4.6.2. Associations of Fetal Overnutrition Typology with Offspring Metabolite Profiles

First, we consolidated the metabolites from Step 1 into distinct metabolite profiles using principal component analysis (PCA), an unsupervised dimension-reduction approach that creates latent variables (i.e., metabolite profiles) based on their intercorrelations. This procedure, completed separately for metabolite data at the childhood and adolescent visit, reduced the number of subsequent comparisons and enhanced interpretability given that metabolites on related metabolic pathways are correlated with one another. Upon creation of the PCA factors, we determined the number of factors to retain at each visit based on visual inspection of the Scree plots. To interpret the factors, we assessed the composition of each factor and focused on metabolites with positive factor loadings ≥0.40.

When assessing the composition of each factor at the childhood and adolescent visits, we found that despite slight differences in the factor order—an indicator of the amount of variation explained by each factor—there were factors with the same combination of high-loading metabolites at both visits. For instance, the high-loading metabolites of the first factor at the childhood and adolescent visit were the same, as was the case for high-loading metabolites of the third factor at the childhood visit and the second factor of the adolescent visit, despite slight variation in the exact degree of loading. These factors, for which metabolite composition was similar across the two visits, were of interest as they represent persistent metabolic differences in the offspring’s metabolite profile that spanned both time points.

We then used linear mixed models to examine associations of fetal overnutrition typology with the metabolite factors across the childhood and adolescent visits. In the models, fetal overnutrition was categorized as a four-level parameter: obesity and GDM, obesity only, GDM only, and neither obesity nor GDM (reference) and repeated metabolite factors were the outcome. We used contrast statements to compare the difference in our response variable (metabolite factors) between different levels of the fetal overnutrition parameter. This allows us to compare the difference in metabolite factor between all three different combinations of our fetal overnutrition parameter. The contrasts used the following specification: (1) obesity and GDM vs. GDM only, (2) obesity and GDM vs. obesity only, and (3) obesity only vs. GDM. If a factor was associated with a specific fetal overnutrition typology, we then further explored differences in individual metabolites from that factor. We assessed unadjusted associations followed by adjusted associations after accounting for maternal prenatal smoking, and offspring sex, age, and ethnicity and race.

The following sensitivity analyses were conducted. Seven women in the GDM-only typology had type-one diabetes; we excluded them in multivariable models to assess if potential etiological differences of hyperglycemia in pregnancy impacted the conclusions. Second, although birthweight, BMI, kilocalories, and physical activity are potential mediators to the relationship between fetal overnutrition and metabolite profiles during childhood and adolescence, we assessed the impact of including birthweight-for-gestational-age z-score, and repeated measures of BMI, kilocalories, and physical activity in sperate multivariable models. Third, in multivariable models we additionally adjusted for Tanner stage at the childhood and adolescent visits. Fourth, we assessed the impact of adjustment for GDM treatment in the multivariable models. Lastly, we tested for an interaction with offspring sex in unadjusted models to assess for evidence that the association between fetal overnutrition and metabolite profiles/factors scores was different in males versus females.

#### 4.6.3. Correlation of Offspring Metabolite Profiles and Indicators of Metabolic Health and Lifestyle

We used Pearson correlation coefficients to inform interpretation of offspring metabolite profiles captured by the factors and their correlation to concurrently measured conventional indicators of metabolic health (conventional metabolic biomarkers, anthropometry, and lifestyle). Statistical analyses were performed with SAS version 9.4 (SAS Institute, Cary, NC, USA).

## Figures and Tables

**Table 1:** Bivariate associations of fetal overnutrition typologies with participant characteristics.

		Overnutrition Typology			
	No GDM or Obesity	Obese Only	GDM Only	GDM & Obesity	*p* ^1^
Maternal Characteristics	*n* = 293	*n* = 66	*n* = 56	*n =* 25	
Pre-pregnancy BMI (kg/m^2^)	23.2 ± 3.1	35.0 ± 5.3	24.0 ± 3.4	35.1 ± 4.1	-
Education level					0.10
<High school	3.1 (9)	6.1 (4)	5.4 (3)	4.0 (1)	
High school or some college	44.4 (130)	62.1 (41)	46.4 (26)	56.0 (14)	
Associates degree or higher	52.6 (154)	31.8 (21)	48.2 (27)	40.0 (10)	
Smoked during pregnancy	7.2 (21)	6.1 (4)	17.9 (10)	20.0 (5)	0.01
Offspring characteristics at birth					
Female	48.8 (143)	47.0 (31)	46.4 (26)	28.0 (7)	0.26
Race/ethnicity					<0.001
Non-Hispanic White	42.3 (124)	18.2 (12)	71.4 (40)	52.0 (13)	
Hispanic	44.0 (129)	57.6 (38)	21.4 (12)	44.0 (11)	
Non-Hispanic Black	7.2 (21)	21.2 (14)	3.6 (2)	4.0 (1)	
Non-Hispanic Other	6.5 (19)	3.0 (2)	3.6 (2)	0.0 (0)	
Birthweight for gestational age z-score ^2^	−0.4 ± 0.9	−0.3 ± 1.0	−0.1 ± 1.0	0.0 ± 0.9	0.02
Childhood visit					
Age, years	10.7 ± 1.4	10.5 ± 1.4	9.5 ± 1.8	9.8 ± 1.5	<0.001
BMI (kg/m^2^)	18.6 ± 4.0	21.1 ± 5.5	18.1 ± 4.2	20.6 ± 5.7	<0.001
BMI z-score	0.2 ± 1.2	0.9 ± 1.1	0.1 ± 1.4	0.8 ± 1.1	<0.001
Kilocalories	1791.5 ± 565.3	1819.8 ± 581.1	1773.0 ± 495.5	1727.4 ± 415.2	0.90
Energy expenditure	68.4 ±11.3	65.15 ± 9.41	66.04 ± 9.39	65.64 ± 11.91	0.09
Adolescent visit					
Age, years	16.7 ± 1.1	16.4 ± 1.3	15.8 ± 1.1	16.0 ± 1.0	<0.001
BMI (kg/m^2^)	22.7 ± 4.8	28.0 ± 7.1	22.6 ± 4.6	24.8 ± 6.3	<0.001
BMI z-score ^3^	0.2 ± 1.1	1.2 ± 1.0	0.4 ± 1.1	0.8 ± 1.1	<0.001
Kilocalories	1672.5 ± 717.0	1599.4 ± 762.3	1730.2 ± 887.6	1660.7 ± 577.5	0.88
Energy expenditure	70.5 ± 16.1	66.87 ± 11.89	67.8 ± 13.5	78.25 ± 20.12	0.05

^1^ ANOVA for continuous variables; Pearson chi-squared test for categorical variables. ^2^ Birthweight for gestational age z-score based on U.S. national reference [13]. ^3^ Age- and sex-specific z scores according to the WHO Growth Reference for children aged 5–19 years [14]. Abbreviations: BMI, Body Mass Index; GDM, Gestational Diabetes Mellitus.

**Table 2:** Metabolites in fasting serum of 440 youth in the Exploring Perinatal Outcomes among Children (EPOCH) cohort across 6 years of follow-up (childhood–adolescences) that differed with respect to exposure to any fetal overnutrition (OB and/or GDM vs. neither).

Compound	Superclass	Subclass	*p*-Value	FDR *p*-Value
Tyrosine	Amino Acid	Tyrosine Metabolism	0.001	0.119
Homoarginine	Amino Acid	Urea cycle; Arginine and Proline Metabolism	0.002	0.144
2-hydroxy-3-methylvalerate	Amino Acid	Leucine, Isoleucine and Valine Metabolism	0.004	0.144
3-methyl-2-oxobutyrate	Amino Acid	Leucine, Isoleucine and Valine Metabolism	0.006	0.144
2-aminoadipate	Amino Acid	Lysine Metabolism	0.007	0.154
Glycine	Amino Acid	Glycine, Serine and Threonine Metabolism	0.007	0.154
N-acetylglycine	Amino Acid	Glycine, Serine and Threonine Metabolism	0.008	0.158
Methionine sulfoxide	Amino Acid	Methionine, Cysteine, SAM, Taurine	0.010	0.167
Alpha-hydroxyisocaproate	Amino Acid	Leucine, Isoleucine and Valine Metabolism	0.014	0.190
Mannitol/sorbitol	Carbohydrate	Fructose, Mannose and Galactose Metabolism	0.003	0.144
Glucuronate	Carbohydrate	Aminosugar Metabolism	0.004	0.144
Mannose	Carbohydrate	Fructose, Mannose and Galactose Metabolism	0.005	0.144
Pantothenate	Cofactors, Vitamins	Pantothenate and CoA Metabolism	0.001	0.119
Alpha-ketoglutarate	Energy	TCA Cycle	0.001	0.119
Citrate	Energy	TCA Cycle	0.004	0.144
Malate	Energy	TCA Cycle	0.006	0.144
Succinate	Energy	TCA Cycle	0.012	0.181
12-HETE	Lipid	Eicosanoid	0.000	0.119
13-HODE + 9-HODE	Lipid	Fatty Acid, Monohydroxy	0.002	0.119
Hydroxy-CMPF ^1^	Lipid	Fatty Acid, Dicarboxylate	0.005	0.144
Choline	Lipid	Phospholipid Metabolism	0.005	0.144
3-hydroxybutyroylglycine ^1^	Lipid	Fatty Acid Metabolism(Acyl Glycine)	0.005	0.144
7-alpha-hydroxy-3-oxo-4-cholestenoate (7-Hoca)	Lipid	Sterol	0.006	0.144
Palmitoyl-arachidonoyl-glycerol (16:0/20:4) [2] ^1^	Lipid	Diacylglycerol	0.006	0.144
N-oleoylserine	Lipid	Endocannabinoid	0.007	0.154
1-linoleoyl-GPA (18:2) ^1^	Lipid	Lysophospholipid	0.009	0.158
Hexanoylcarnitine (C6)	Lipid	Fatty Acid Metabolism(Acyl Carnitine)	0.009	0.161
3-carboxy-4-methyl-5-propyl-2-furanpropanoate (CMPF)	Lipid	Fatty Acid, Dicarboxylate	0.012	0.180
Glycosyl-N-palmitoyl-sphingosine (d18:1/16:0)	Lipid	Hexosylceramides (HCER)	0.012	0.181
1-(1-enyl-palmitoyl)-GPC (P-16:0) ^1^	Lipid	Lysoplasmalogen	0.013	0.188
Sphingomyelin (d18:2/14:0, d18:1/14:1) ^1^	Lipid	Sphingomyelins	0.014	0.190
Dodecadienoate (12:2) ^1^	Lipid	Fatty Acid, Dicarboxylate	0.016	0.193
1-(1-enyl-palmitoyl)-2-oleoyl-GPE (P-16:0/18:1) ^1^	Lipid	Plasmalogen	0.016	0.193
Sphingomyelin (d18:0/18:0, d19:0/17:0) ^1^	Lipid	Dihydrosphingomyelins	0.016	0.193
Dihydroorotate	Nucleotide	Pyrimidine Metabolism, Orotate contain.	0.002	0.119
Urate	Nucleotide	Purine Metabolism, (Hypo)Xanthine/Inosine	0.009	0.158
N1-methyladenosine	Nucleotide	Purine Metabolism, Adenine contain.	0.010	0.167
Guanosine	Nucleotide	Purine Metabolism, Guanine contain.	0.013	0.189
Fibrinopeptide A, des-ala(1) ^1^	Peptide	Fibrinogen Cleavage Peptide	0.001	0.119
Gamma-glutamylglutamate	Peptide	Gamma-glutamyl Amino Acid	0.004	0.144
Gamma-glutamylcitrulline ^1^	Peptide	Gamma-glutamyl Amino Acid	0.004	0.144
Gamma-glutamyl-alpha-lysine	Peptide	Gamma-glutamyl Amino Acid	0.005	0.144
Glycylvaline	Peptide	Dipeptide	0.008	0.158
Gamma-glutamylthreonine	Peptide	Gamma-glutamyl Amino Acid	0.009	0.158
Gamma-glutamyl-2-aminobutyrate	Peptide	Gamma-glutamyl Amino Acid	0.009	0.163
Gamma-glutamylglycine	Peptide	Gamma-glutamyl Amino Acid	0.014	0.190
Phenylalanylglycine	Peptide	Dipeptide	0.015	0.193
Sulfate of piperine metabolite C16H19NO3 (2) ^1^	Xenobiotics	Food Component/Plant	0.000	0.119
Sulfate of piperine metabolite C16H19NO3 (3) ^1^	Xenobiotics	Food Component/Plant	0.001	0.119
Quinate	Xenobiotics	Food Component/Plant	0.004	0.144
Piperine	Xenobiotics	Food Component/Plant	0.004	0.144
Perfluorooctanesulfonate (PFOS)	Xenobiotics	Chemical	0.011	0.174

Abbreviations: OB, pre-pregnancy obesity; GDM, Gestational Diabetes Mellitus. Adjusted for maternal smoking in pregnancy, child sex, race, and age at visit. ^1^ Tier 2 identification in which no commercially available authentic standards could be found, but annotated based on accurate mass, spectral and chromatographic similarity to tier 1-identified compounds.

**Table 3:** Metabolite factors and factor loadings of the top metabolites at the childhood and adolescent visits.

Factor Loading				
Childhood Visit	Adolescent Visit	Compound	Superclass	Subclass
Factor label: γ-glutamyl				
Factor 1 (44% variance)	Factor 1 (20% variance)			
0.81	0.83	Gamma-glutamylglutamate	Peptide	Gamma-glutamyl Amino Acid
0.77	0.81	Gamma-glutamyl-alpha-lysine	Peptide	Gamma-glutamyl Amino Acid
0.72	0.69	Gamma-glutamylglycine	Peptide	Gamma-glutamyl Amino Acid
0.70	0.74	Methionine sulfoxide	Amino Acid	Methionine, Cysteine, SAM, Taurine Metabolism
0.66	0.68	Glycylvaline	Peptide	Dipeptide
0.66	0.68	1-linoleoyl-GPA (18:2)	Lipid	Lysophospholipid
0.61	<0.40	13-HODE + 9-HODE	Lipid	Fatty Acid, Monohydroxy
0.61	0.65	Choline	Lipid	Phospholipid Metabolism
<0.40	0.62	Gamma-glutamylthreonine	Peptide	Gamma-glutamyl Amino Acid
Factor label: Sphingomyelin-mannose				
Factor 3 ^a^ (8% variance)	Factor 2 ^a^ (13% variance)			
0.61	0.58	Sphingomyelin (d18:2/14:0, d18:1/14:1)	Lipid	Sphingomyelins
0.59	0.64	Sphingomyelin (d18:0/18:0, d19:0/17:0)	Lipid	Dihydrosphingomyelins
0.54	0.59	Mannose	Carbohydrate	Fructose, Mannose and Galactose Metabolism
0.52	0.58	Homoarginine	Amino Acid	Urea cycle; Arginine and Proline Metabolism
0.45	0.50	N1-methyladenosine	Nucleotide	Purine Metabolism, Adenine containing
Factor label: Skeletal muscle metabolism				
Factor 4 (6% variance)	Factor 4 (10% variance)			
0.63	0.76	Alpha-hydroxyisocaproate	Amino Acid	Leucine, Isoleucine and Valine Metabolism
0.49	0.62	2-hydroxy-3-methylvalerate	Amino Acid	Leucine, Isoleucine and Valine Metabolism
0.40	0.51	Malate	Energy	TCA Cycle
0.40	0.51	Urate	Nucleotide	Purine Metabolism, (Hypo)Xanthine/Inosine
0.40	<0.40	Citrate	Energy	TCA Cycle
<0.40	0.41	7-alpha-hydroxy-3-oxo-4-cholestenoate (7-Hoca)	Lipid	Sterol
Factor label: 3-carboxy-4-methyl-5-propyl-2-furanpropanoic acid (CMPF)				
Factor 6 (3% variance)	Factor 6 (4% variance)			
0.74	0.91	3-carboxy-4-methyl-5-propyl-2-furanpropanoate (CMPF)	Lipid	Fatty Acid, Dicarboxylate
0.71	0.90	Hydroxy-CMPF	Lipid	Fatty Acid, Dicarboxylate
Factor 2 ^a^ (11% variance)	Factor 3 ^a^ (13% variance)			
0.58	0.94	Sulfate of piperine metabolite C16H19NO3 (2)	Xenobiotics	Food Component/Plant
0.56	0.93	Sulfate of piperine metabolite C16H19NO3 (3)	Xenobiotics	Food Component/Plant
0.51	0.85	Piperine	Xenobiotics	Food Component/Plant
0.50	<0.40	2-aminoadipate	Amino Acid	Lysine Metabolism
0.45	<0.40	2-hydroxy-3-methylvalerate	Amino Acid	Leucine, Isoleucine and Valine Metabolism
Factor 5 (4% variance)	Factor 5 (4% variance)			
0.50	<0.40	Sulfate of piperine metabolite C16H19NO3 (2)	Xenobiotics	Food Component/Plant
0.49	<0.40	Sulfate of piperine metabolite C16H19NO3 (3)	Xenobiotics	Food Component/Plant
0.43	<0.40	Piperine	Xenobiotics	Food Component/Plant
<0.40	0.53	Dodecadienoate (12:2) *	Lipid	Fatty Acid, Dicarboxylate
<0.40	0.53	3-hydroxybutyroylglycine *	Lipid	Fatty Acid Metabolism (Acyl Glycine)
<0.40	0.44	Hexanoylcarnitine (C6)	Lipid	Fatty Acid Metabolism (Acyl Carnitine)
<0.40	0.57	N-acetylglycine	Amino Acid	Glycine, Serine and Threonine Metabolism
<0.40	0.45	Glycine	Amino Acid	Glycine, Serine and Threonine Metabolism

Note: Labeled factors are of interest due to similarity in non-xenobiotic composition at both the childhood and adolescent visits. ^a^ Factor number differed across research visits. * Tier 2 identification in which no commercially available authentic standards could be found, but annotated based on accurate mass, spectral and chromatographic similarity to tier 1-identified compounds.

**Table 4:** Longitudinal associations (β [95% CI]) of fetal overnutrition (obesity and GDM, obesity only, and GDM only) with metabolite factor scores across 6 years of follow-up among 444 youth in the EPOCH cohort.

	OB + GDM vs. GDM Only	OB + GDM vs. OB Only	OB Only vs. GDM Only
Factor	Adjusted	Adjusted	Adjusted
γ-glutamyl	−0.20 (−0.50, 0.10)	−0.14 (−0.44, 0.17)	−0.06 (−0.32, 0.20)
Sphingomyelin-mannose	0.29 (−0.04, 0.63)	−0.03 (−0.38, 0.33)	0.32 (0.07, 0.57) *
Skeletal muscle metabolism	0.36 (0.09, 0.64) *	0.47 (0.21, 0.72) *	−0.10 (−0.34, 0.13)
CMPF	0.50 (0.11, 0.89) *	0.05 (−0.34, 0.44)	0.45 (0.17, 0.73) *

Adjusted model includes maternal smoking in pregnancy, child sex, race, and age at visit. Abbreviations: CMPF, 3-carboxy-4-methyl-5-propyl-2-furanpropanoic acid; GDM, Gestational Diabetes Mellitus OB, pre-pregnancy obesity. * *p* < 0.05.

## Data Availability

Data Availability Statement: Because of the participant consent obtained as part of the recruitment process, it is not possible to make these data publicly available. The data resented in this study are available on request from the corresponding author.

## Supplementary Materials

The following supporting information can be downloaded at: https://www.mdpi.com/article/10.3390/metabo12030265/s1, Table S1: Longitudinal associations (β [95% CI]) of fetal overnutrition (obesity and GDM, obesity only, and GDM only) with top loading metabolites across 6 years of follow-up among 444 youth in the EPOCH cohort, Table S2: Adjusted longitudinal associations (β [95% CI]) of fetal overnutrition (obesity and GDM, obesity only, and GDM only) with metabolite factor scores across 6 years of follow-up among 444 youth in the EPOCH cohort.
