# Supplementary material for: Metabolomic Profiles in Childhood and Adolescence Are Associated with Fetal Overnutrition
Source: Metabolites. 2022 Mar 19;12(3):265. doi: 10.3390/metabo12030265 (PMC8952572; doi:10.3390/metabo12030265)
Supplement: Supplementary file 1 [file metabolites-12-00265-s001.zip › metabolites-1553618-supplementary.pdf]

Supplementary Material

Title: Metabolomic profiles in childhood and adolescence associated with fetal overnutrition

Authors: Ellen C. Francis; Katerina Kechris; Catherine C. Cohen; Gregory Michelotti; Dana Dabelea; Wei Perng

Supplementary Table S1: Longitudinal associations (β [95% CI]) of fetal overnutrition (obesity and GDM, obesity only, and GDM only) with top loading metabolites across 6 years of follow-up among 444 youth in the EPOCH cohort.

Factor loading by visit childhood	Factor loading by visit adolescent	Compound	Superclass	Subclass	OB + GDM vs. GDM only	OB + GDM vs. OB only	OB only vs. GDM only
		**Sphingomyelin-mannose**					
0.61	0.58	Sphingomyelin (d18:2/14:0, d18:1/14:1)	Lipid	Sphingomyelins	0.03 (-0.03, 0.09)	0.01 (-0.06, 0.07)	0.03 (-0.01, 0.06)
0.59	0.64	Sphingomyelin (d18:0/18:0, d19:0/17:0)	Lipid	Dihydrosphingomyelins	0.03 (-0.07, 0.13)	0.00 (-0.11, 0.10)	0.03 (-0.04, 0.10)
0.54	0.59	Mannose	Carbohydrate	Fructose, Mannose and Galactose Metabolism	0.04 (-0.02, 0.10)	-0.01 (-0.07, 0.04)	0.06 (0.00, 0.11) *
0.52	0.58	Homoarginine	Amino Acid	Urea cycle; Arginine and Proline Metabolism	0.07 (0.02, 0.11) *	0.04 (-0.01, 0.09)	0.03 (-0.01, 0.07)
0.45	0.50	N1-methyladenosine	Nucleotide	Purine Metabolism, Adenine containing	0.01 (-0.05, 0.07)	0.01 (-0.05, 0.07)	0.00 (-0.04, 0.05)
		**Skeletal muscle metabolism**					
0.63	0.76	Alpha-hydroxyisocaproate	Amino Acid	Leucine, Isoleucine and Valine Metabolism	-0.01 (-0.05, 0.03)	0.02 (-0.02, 0.06)	-0.03 (-0.07, 0.01)
0.49	0.62	2-hydroxy-3-methylvalerate	Amino Acid	Leucine, Isoleucine and Valine Metabolism	0.01 (-0.04, 0.06)	0.03 (-0.02, 0.09)	-0.02 (-0.07, 0.02)
0.40	0.51	Malate	Energy	TCA Cycle	0.00 (-0.05, 0.05)	0.02 (-0.03, 0.07)	-0.02 (-0.06, 0.02)
0.40	0.51	Citrate	Energy	TCA Cycle	0.00 (-0.05, 0.04)	0.02 (-0.03, 0.06)	-0.02 (-0.06, 0.02)
0.40	<.40	Urate	Nucleotide	Purine Metabolism, (Hypo)Xanthine/Inosine	0.04 (0.00, 0.07) *	0.04 (0.00, 0.07) *	0.00 (-0.03, 0.03)
<.40	0.41	7-alpha-hydroxy-3-oxo-4-cholestenoate (7-Hoca)	Lipid	Sterol	0.01 (-0.03, 0.06)	0.05 (0.00, 0.09) *	-0.03 (-0.07, 0.00)
		**CMPF**					
0.74	0.91	3-carboxy-4-methyl-5-propyl-2-furanpropanoate (CMPF)	Lipid	Fatty Acid, Dicarboxylate	0.27 (0.06, 0.48) *	0.07 (-0.15, 0.29)	0.20 (0.05, 0.34) *
0.71	0.90	Hydroxy-CMPF	Lipid	Fatty Acid, Dicarboxylate	0.17 (-0.03, 0.37)	-0.01 (-0.21, 0.20)	0.18 (0.03, 0.33) *

Adjusted for maternal smoking in pregnancy, child sex, race, and age at visit.

Abbreviations: CMPF, 3-carboxy-4-methyl-5-propyl-2-furanpropanoic acid; GDM, Gestational Diabetes Mellitus OB, prepregnancy obesity

* P<.05

Supplementary Table S2: Adjusted longitudinal associations (β [95% CI]) of fetal overnutrition (obesity and GDM, obesity only, and GDM only) with metabolite factor scores across 6 years of follow-up among 444 youth in the EPOCH cohort.

Factors	OB + GDM vs. GDM only	OB + GDM vs. OB only	OB only vs. GDM only
**Model A (Tanner stage at each visit)**			
γ-glutamyl	-0.21 (-0.52, 0.10)	-0.18 (-0.51, 0.14)	-0.03 (-0.29, 0.23)
Sphingomyelin-mannose	0.31 (-0.04, 0.65)	0.00 (-0.36, 0.37)	0.30 (0.04, 0.56) *
Skeletal muscle metabolism	0.35 (0.07, 0.63) *	0.46 (0.21, 0.72) *	-0.11 (-0.35, 0.12)
CMPF	0.51 (0.12, 0.90) *	0.06 (-0.33, 0.44)	0.45 (0.17, 0.73) *
**Model B (BMI at each visit)**			
γ-glutamyl	-0.18 (-0.48, 0.12)	-0.14 (-0.44, 0.17)	-0.05 (-0.31, 0.22)
Sphingomyelin-mannose	0.20 (-0.12, 0.52)	-0.01 (-0.34, 0.31)	0.21 (-0.03, 0.45)
Skeletal muscle metabolism	0.32 (0.06, 0.59) *	0.48 (0.24, 0.71) *	-0.15 (-0.39, 0.08)
CMPF	0.52 (0.12, 0.91) *	0.05 (-0.34, 0.44)	0.47 (0.18, 0.75) *
**Model C (Kcal at each visit)**			
γ-glutamyl	-0.21 (-0.51, 0.10)	-0.15 (-0.45, 0.16)	-0.06 (-0.32, 0.20)
Sphingomyelin-mannose	0.31 (-0.03, 0.65)	-0.03 (-0.40, 0.33)	0.34 (0.09, 0.60) *
Skeletal muscle metabolism	0.37 (0.08, 0.65) *	0.46 (0.20, 0.72) *	-0.09 (-0.34, 0.15)
CMPF	0.53 (0.14, 0.92) *	0.07 (-0.33, 0.46)	0.46 (0.18, 0.74) *
**Model D (physical activity at each visit)**			
γ-glutamyl	-0.22 (-0.52, 0.09)	-0.15 (-0.46, 0.17)	-0.07 (-0.34, 0.19)
Sphingomyelin-mannose	0.37 (0.06, 0.69) *	0.02 (-0.31, 0.36)	0.35 (0.09, 0.61) *
Skeletal muscle metabolism	0.41 (0.13, 0.69) *	0.48 (0.21, 0.74) *	-0.07 (-0.31, 0.17)
CMPF	0.54 (0.16, 0.92) *	0.09 (-0.29, 0.46)	0.45 (0.17, 0.73) *
**Model E (birthweight z-score)**			
γ-glutamyl	-0.20 (-0.50, 0.10)	-0.15 (-0.46, 0.15)	-0.05 (-0.31, 0.21)
Sphingomyelin-mannose	0.29 (-0.05, 0.63)	-0.03 (-0.39, 0.33)	0.32 (0.07, 0.58) *
Skeletal muscle metabolism	0.37 (0.10, 0.65) *	0.50 (0.24, 0.75) *	-0.12 (-0.36, 0.11)
CMPF	0.50 (0.11, 0.89) *	0.05 (-0.35, 0.44)	0.45 (0.17, 0.74) *

Model A: Adjusted for maternal smoking in pregnancy, child sex, race, and age and Tanner stage at visit.

Model C: Adjusted for maternal smoking in pregnancy, child sex, race, and age and kilocalories at visit.

Model D: Adjusted for maternal smoking in pregnancy, child sex, race, and age and mean physical activity at visit.

Model E: Adjusted for maternal smoking in pregnancy, child sex, race, birthweight-for-gestational-age z-score, and age at visit.

Abbreviations: CMPF, 3-carboxy-4-methyl-5-propyl-2-furanpropanoic acid; GDM, Gestational Diabetes Mellitus OB, prepregnancy obesity

* P<.05
